# Centromeric transcription maintains centromeric cohesion in human cells

**DOI:** 10.1083/jcb.202008146

**Published:** 2021-04-21

**Authors:** Yujue Chen, Qian Zhang, Zhen Teng, Hong Liu

**Affiliations:** 1 Department of Biochemistry and Molecular Biology, Tulane University School of Medicine, New Orleans, LA; 2 Tulane Cancer Center, Tulane University School of Medicine, New Orleans, LA; 3 Tulane Aging Center, Tulane University School of Medicine, New Orleans, LA

## Abstract

Centromeric transcription has been shown to play an important role in centromere functions. However, lack of approaches to specifically manipulate centromeric transcription calls into question that the proposed functions are a direct consequence of centromeric transcription. By monitoring nascent RNAs, we found that several transcriptional inhibitors exhibited distinct, even opposing, efficacies on the suppression of ongoing gene and centromeric transcription in human cells, whereas under the same conditions, total centromeric RNAs were changed to a lesser extent. The inhibitor suppressing ongoing centromeric transcription weakened centromeric cohesion, whereas the inhibitor increasing ongoing centromeric transcription strengthened centromeric cohesion. Furthermore, expression of CENP-B DNA-binding domain or CENP-B knockdown moderately increased centromeric transcription without altering gene transcription; as a result, centromeric cohesion was accordingly strengthened. Targeting of the Kox1-KRAB domain with CENP-B DB to centromeres specifically decreased centromeric transcription and weakened centromeric cohesion. Thus, based on these findings, we propose that a major function of centromeric transcription is to maintain centromeric cohesion in human cells.

## Teaser

Centromeric transcription is critical for proper centromere function, but its exact role remains elusive. By manipulating the activity of centromeric transcription, Chen et al. report that a major function of centromeric transcription in human cells is to maintain centromeric cohesion.

## Introduction

The centromere is the specialized DNA sequence of a chromosome that dictates the assembly of kinetochores during cell division, which is essential for proper chromosome segregation. In most eukaryotes, centromeric DNA contains tandemly repetitive sequences that usually do not encode any proteins. These DNA repeats were historically considered heterochromatic and thereby transcriptionally inert; but increasing evidence suggests that they are under active transcription mainly performed by RNA polymerase (RNAP) II (Hall et al., 2012). It has been accepted that centromeric transcription plays an important role in proper centromere functions (Mehta et al., 2010; Smurova and De Wulf, 2018). Ongoing transcription and/or centromeric transcripts were reported to promote the deposition of CENP-A, a variant of histone H3 that defines centromeres, to centromeric chromatin in various types of eukaryotes, including fission yeast, fruit fly, and human (Bobkov et al., 2018; Bobkov et al., 2020; Chen et al., 2015; Choi et al., 2012; Folco et al., 2008; McNulty et al., 2017; Quénet and Dalal, 2014; Rošić et al., 2014; Swartz et al., 2019). It has also been shown that centromeric transcripts are able to bind various centromere proteins (Blower, 2016; Du et al., 2010; Ferri et al., 2009; Jambhekar et al., 2014; Quénet and Dalal, 2014; Rošić et al., 2014; Topp et al., 2004; Wong et al., 2007), presumably regulating the functions of these proteins. In addition, centromeric transcription of human cells may also promote centromeric cohesion at early mitosis and proper chromosome segregation during anaphase (Chan et al., 2012; Liu et al., 2015).

In some of these studies, general transcriptional inhibitors were applied to suppress centromeric transcription. For example, treatment of triptolide and THZ1, which both inhibit the transcriptional initiation factor TFIIH, decreased the deposition of newly synthesized CENP-A into the centromeric chromatin in fruit fly and starfish cells (Bobkov et al., 2018; Swartz et al., 2019). In mitosis, treatment of human cells with α-amanitin, a small cyclic peptide that directly binds RNAP II and inhibits its elongation, induced a significant increase in centromeric cohesion defects and anaphase lagging chromosomes (Chan et al., 2012; Liu et al., 2015). Surprisingly, treatment of mitotic human cells with triptolide did not yield the similar defects that were observed in cells treated with α-amanitin (Novais-Cruz et al., 2018; Perea-Resa et al., 2020). These seemingly inconsistent results may simply suggest differential efficacies of these inhibitors on the suppression of centromeric transcription in distinct types of cells. Nevertheless, because the efficacies of these inhibitors were not rigorously measured in these studies, it is unknown whether centromeric transcription was effectively suppressed. Alternatively, it is possible that the inhibitor-induced phenotypes might not be a direct consequence of suppressed centromeric transcription, as these transcriptional inhibitors suppress transcription globally. Hence, it is critically important to develop novel approaches to specifically inactivate centromeric transcription without altering global gene transcription so that the functions of centromeric transcription can be accurately determined.

In the present study, we found that general transcriptional inhibitors exhibited distinct, even opposing, efficacies on the suppression of centromeric transcription. The inhibitor suppressing ongoing centromeric transcription weakened centromeric cohesion in mitotic cells, whereas the one increasing ongoing centromeric transcription strengthened centromeric cohesion. Furthermore, using CENP-B (centromere protein B) DB (DNA-binding domain), we targeted the transcriptional suppressor Kox1 specifically to centromeres, which decreased centromeric transcription and weakened centromeric cohesion in mitotic cells. Thus, based on these findings, we propose that a major function of centromeric transcription is to maintain centromeric cohesion in human cells.

## Results and discussion

### Transcriptional inhibitors exhibit distinct, even opposing, efficacies on the suppression of ongoing α-satellite and gene transcription

**Figure 1.**
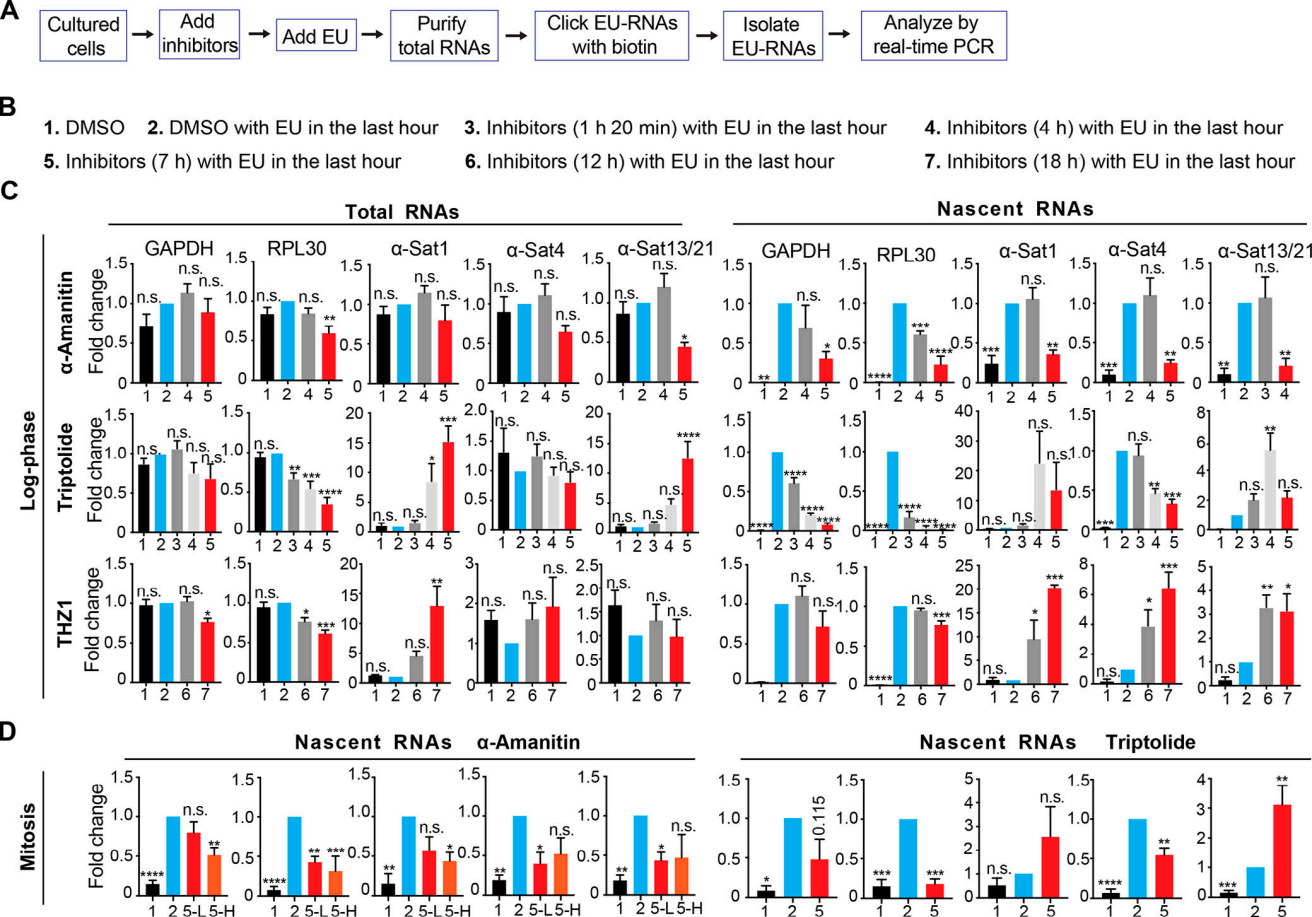
**Transcriptional inhibitors exhibit distinct, even opposing, efficacies on the suppression of ongoing α-satellite and gene transcription. (A)** A flow chart illustrating the preparation and analysis of EU-labeled nascent RNAs. Cultured cells are referred to as log-phase (C) or nocodazole-arrested mitotic (D) cells. **(B)** Experimental conditions that were used in C. The times listed here refer to the durations of inhibitor treatment. The inhibitor concentrations were selected based on the toxicity of each inhibitor on the tested human cells. They were near but lower than lethal concentrations. No more than 20% of dead cells were observed under each of the following conditions: α-amanitin: 50 µg/ml, triptolide: 1.4 µM, flavopiridol: 1.0 µM, and THZ1: 120 nM. Treatment of these inhibitors seemed not to significantly alter the cell cycle profile of cultured log-phase (C) or mitotic (D) cells. EU was usually added 1 h before cell harvest. **(C)** HeLa Tet-On cells were treated with various types of transcriptional inhibitors as described in B. **(D)** Nocodazole-arrested mitotic HeLa Tet-On cells were treated with DMSO, α-amanitin (5-L, 5 µg/ml; 5-H, 50 µg/ml) or triptolide for 7 h with EU treatment in the last hour. EU-RNAs were prepared and analyzed by real-time PCR. The details are recorded in the Materials and methods. The average and standard error calculated from at least three independent experiments are shown here. *, P < 0.05; **, P < 0.01; ***, P < 0.001; ****, P < 0.0001. α-Sat, α-satellite.

**Figure S1.**
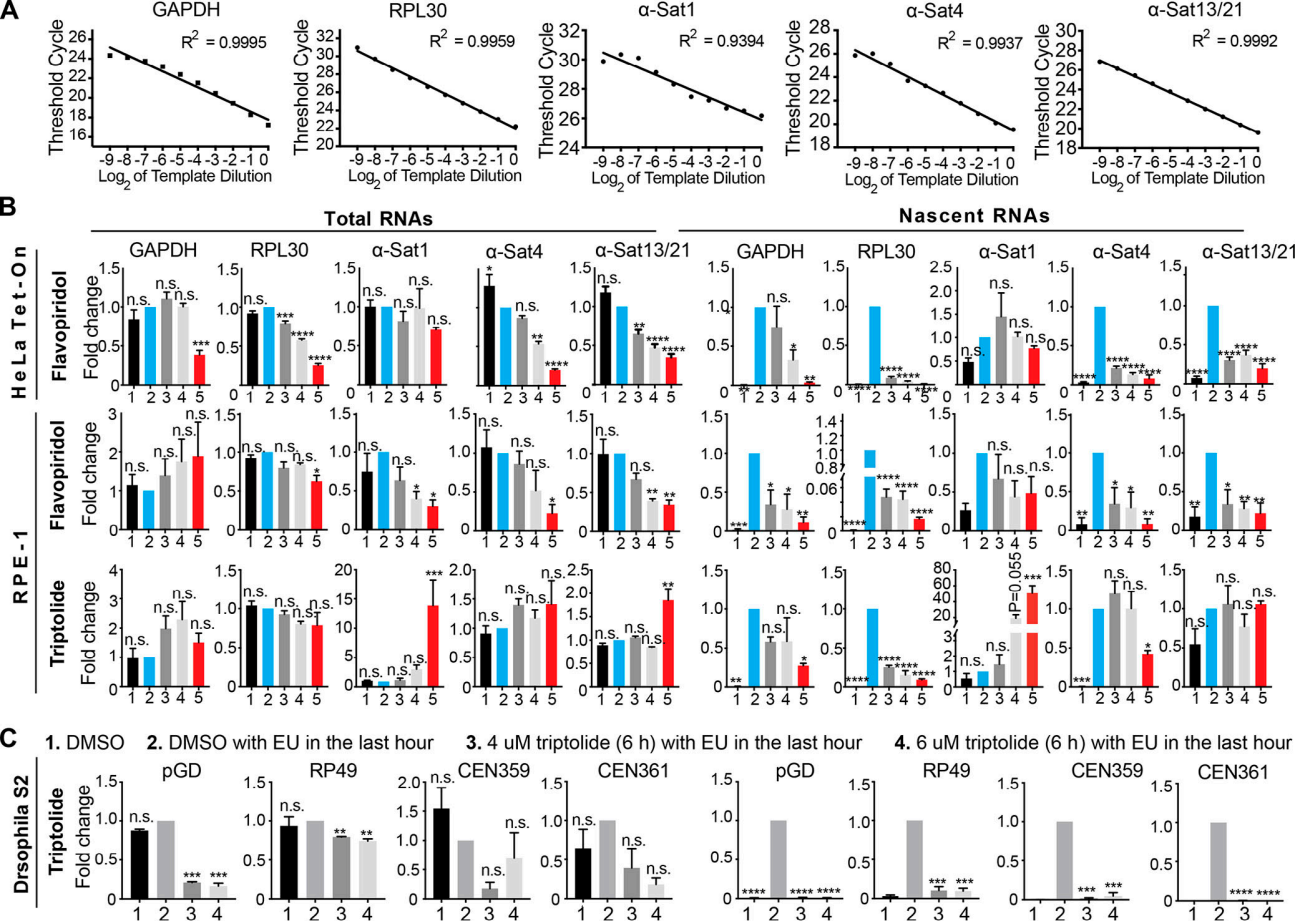
**Efficacies of transcriptional inhibitors on the suppression of gene and centromeric transcription in HeLa Tet-On, RPE-1, and *Drosophila* S2 cells. (A)** Validation of primers. Serially diluted RNA templates (2×) were subjected to real-time PCR analysis with the indicated primers. Ct values were plotted to template dilution, and R^2^ (R, correlation coefficient) was calculated. Scatter plot with linear regression is shown here. **(B)** HeLa Tet-On and RPE-1 cells were treated with the indicated conditions (Fig. 1 B), and total RNAs and EU-RNAs were extracted and purified for real-time PCR analysis with the indicated primers. The average and standard error were calculated based on three independent experiments. **(C)** S2 cells were treated with the indicated conditions, and total RNAs and EU-RNAs were extracted and purified for real-time PCR analysis with the indicated primers. The average and standard error were calculated based on two independent experiments. *, P < 0.05; **, P < 0.01; ***, P < 0.001; ****, P < 0.0001. α-Sat, α-satellite.

The human centromere comprises highly repetitive α-satellite DNA sequences that are transcribed by RNAPII (Talbert and Henikoff, 2018). Because of abundant α-satellite RNAs stored in human nucleoli (Wong et al., 2007), it seems impractical to monitor a rapid change in centromeric transcription within a short period of time by measuring the amount of total α-satellite RNAs. 5′-ethynyl uridine (EU) is an analogue of uridine. It can incorporate into RNAs that are being transcribed. By clicking EU with biotins, these nascent RNAs can be purified and quantified by real-time PCR analysis (Fig. 1 A). Thus, by measuring the amount of EU-labeled nascent α-satellite RNAs, the efficacy of a given transcriptional inhibitor on the suppression of centromeric transcription can be determined. Using this assay, we first evaluated the efficacies of two commonly used transcriptional inhibitors on the suppression of centromeric transcription: α-amanitin (RNAPII elongation inhibitor) and triptolide (TFIIH inhibitor; Bensaude, 2011). Log-phase HeLa Tet-On cells were treated with the inhibitors under different conditions (Fig. 1 B), and EU-RNAs were purified for real-time PCR analysis using two pairs of gene primers, GAPDH and RPL30, and three pairs of centromere primers, α-1, α-4, and α-13/21. These primers were validated for accurate quantification (Fig. S1 A). As α-amanitin is a slow-uptake drug, it started to obviously decrease the amounts of EU-RNAs for GAPDH and RPL30 at 4 h after treatment and to decrease centromeric EU-RNAs at 7 h after treatment (Fig. 1 C). These results are consistent with our previous findings that α-amanitin suppressed centromeric transcription in transcription run-on experiments (Liu et al., 2015; Palozola et al., 2017a; Palozola et al., 2017b). Thus, α-amanitin is competent to suppress centromeric transcription. Triptolide started to decrease the amounts of EU-RNAs for GAPDH and RPL30 as early as 1 h 20 min after treatment, but its effects on centromeric transcription were complex: triptolide moderately decreased the amounts of α-4 EU-RNAs at 4 h and 7 h after treatment but increased the amounts of α-1 and α-13/21 EU-RNAs. These results indicate that triptolide is at least not an effective inhibitor for centromeric transcription, albeit it is for gene transcription. Moreover, α-amanitin and triptolide had similar effects on centromeric transcription in nocodazole-arrested mitotic cells as they did in interphase cells (Fig. 1 D). α-Amanitin at a low concentration of 5 µg/ml worked as effectively as at a high concentration of 50 µg/ml. Thus, α-amanitin, not triptolide, is a competent inhibitor to centromeric transcription.

**Figure S2.**
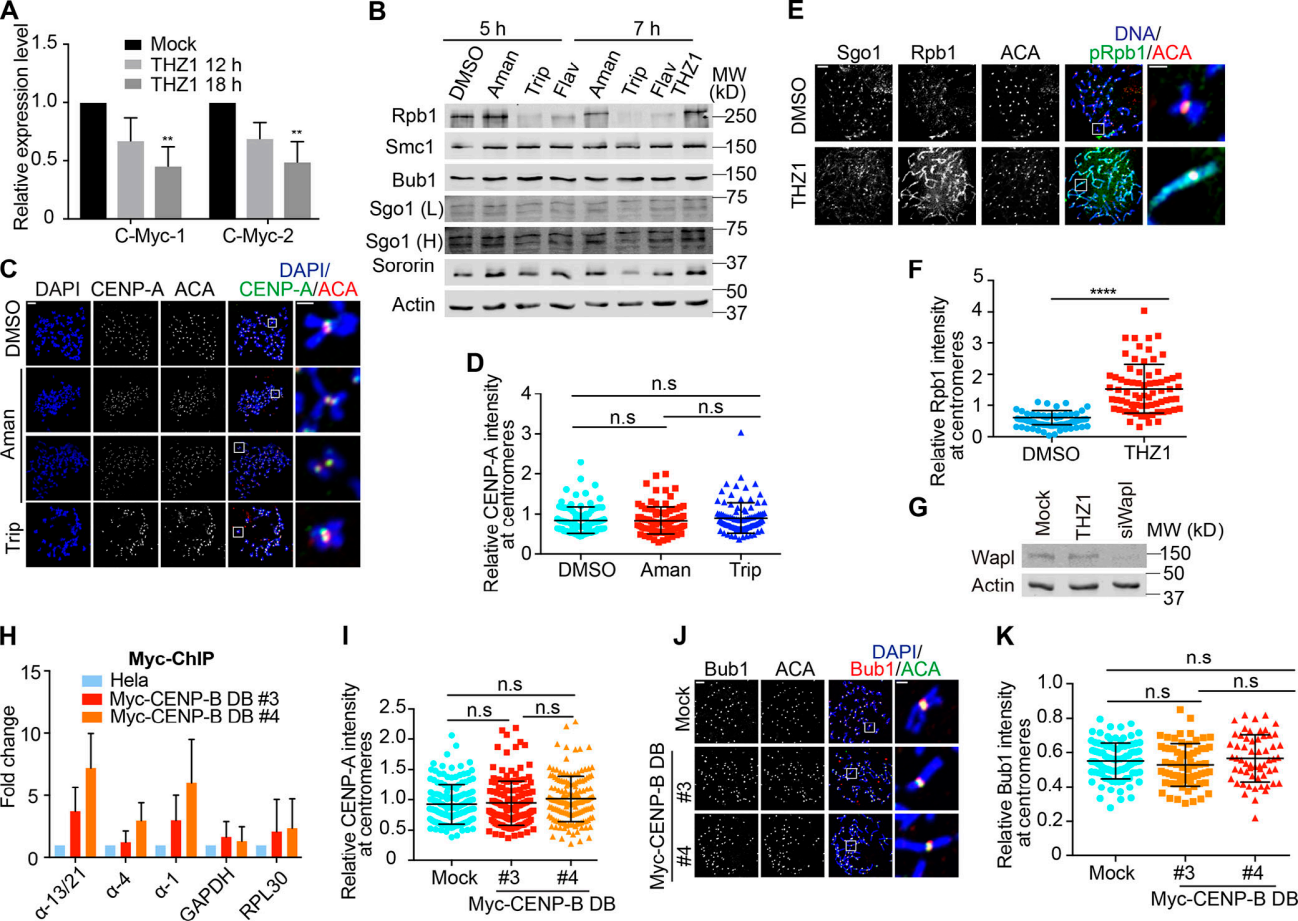
**Treament of transcriptional inhibitors does not change CENP-A levels at centromeres and expression of CENP-B DB does not alter Bub1 levels at centromeres. (A)** RNA extracted from HeLa Tet-On cells with mock treatment or treated with THZ1 was subjected to real-time PCR analysis with the indicated primers. The average and standard error were calculated based on three independent experiments. **(B)** Lysates of HeLa Tet-On cells treated with DMSO, α-amanitin (Aman), triptolide (Trip), flavopiridol (Flav), or THZ1 were subjected to Western blots with the indicated antibodies. L, light exposure; H, heavy exposure. **(C)** Nocodazole-arrested HeLa Tet-On cells treated with DMSO, α-amanitin (Aman), or triptolide (Trip) were subjected to chromosome spread and stained with the indicated antibodies. The scale bars in the left and right panels represent 5 µm and 1 µm, respectively. **(D)** Quantification for relative CENP-A intensity (CENP-A/DNA) at centromeres in C. Quantification details are recorded in the Materials and methods. The experiment was repeated twice, and the results are highly reproducible. Quantification was performed based on the results from a single experiment. The average and standard deviation are shown here. At least 90 centromeres (six per cell) were scored for each condition. **(E)** Thymidine-arrested HeLa Tet-On cells were released into fresh medium containing DMSO or THZ1 and incubated for 12 h with the treatment with nocodazole in the last 2 h. Mitotic cells were collected and subjected to chromosome spread and immunostaining with the indicated antibodies. The scale bars in the left and right panels represent 5 µm and 1 µm, respectively. **(F)** Quantification for relative Rpb1 intensity (Rpb1/ACA) at centromeres in E. The experiment was repeated three times, and the results are highly reproducible. Quantification was performed based on the results from a single experiment. The average and standard deviation are shown here. At least 90 centromeres (six per cell) were scored for each condition. **(G)** Lysates of HeLa Tet-On cells treated with DMSO (12 h), THZ1 (12 h), or siWapl (24 h) were resolved with SDS-PAGE and blotted with the indicated antibodies. **(H)** Nocodazole-arrested HeLa Tet-On cells and HeLa Myc–CENP-B DB stable cells treated with doxycycline were cross-linked with formaldehyde, sonicated, and then subjected to immunoprecipitation with anti-Myc antibodies. DNA extracted from the immunoprecipitates was subjected to real-time PCR analysis with the indicated primers. The average and standard error were calculated based on three independent experiments. **(I)** HeLa Tet-On cells and HeLa Myc–CENP-B DB stable cells treated with doxycycline were incubated with nocodazole for 2 h. Mitotic cells were then subjected to chromosome spread and stained with DAPI and CENP-A antibodies. Quantification of relative CENP-A intensity (CENP-A/DAPI) is shown here. The experiment was repeated twice, and the results are highly reproducible. Quantification was performed based on the results from a single experiment. The average and standard deviation are shown here. At least 90 centromeres (six per cell) were scored for each condition. **(J)** HeLa Tet-On cells and HeLa Myc–CENP-B DB stable cells treated with doxycycline were incubated with nocodazole for 2 h. Mitotic cells were collected for chromosome spread and stained with DAPI, ACA, and anti-Bub1 antibodies. The scale bars in the left and right panels represent 5 µm and 1 µm, respectively. **(K)** Quantification for relative Bub1 intensity (Bub1/ACA) on kinetochores. The experiment was repeated twice, and the results are highly reproducible. Quantification was performed based on the results from a single experiment. The average and standard deviation are shown here. At least 90 centromeres (six per cell) were scored for each condition. **, P < 0.01; ****, P < 0.0001. Mock in A and G denotes DMSO. Mock in I, J, and K, represents parental HeLa Tet-On cells.

We next tested the effects of another pair of transcriptional inhibitors on centromeric transcription: flavopiridol (Cdk9 inhibitor) and THZ1 (Cdk7 inhibitor). Flavopiridol significantly suppressed both the gene and centromeric transcriptions, whereas the effects of THZ1 were distinct. It slightly decreased gene transcription but increased centromeric transcription (Fig. 1 C, Fig. S1 B, and Fig. S2 A; Kwiatkowski et al., 2014). These results suggest that THZ1, instead of being a transcriptional inhibitor, is actually an enhancer for centromeric transcription. The underlying mechanism whereby THZ1 induces centromeric transcription is unknown. Similar results for triptolide and flavopiridol were also obtained in nontransformed RPE-1 cells (Fig. S1 B). Notably, as α-satellite DNA sequences are also present in pericentromeres, changes in EU-RNAs observed here might also be partially contributed by pericentromeric transcription (Johnson et al., 2017). Taken together, although general transcriptional inhibitors can effectively suppress gene transcription, they behave very differently, sometimes opposingly, on centromeric transcription in human cells. The RNAPII elongation inhibitors (α-amanitin and flavopiridol) effectively suppress centromeric transcription, whereas the RNAPII initiation inhibitors (triptolide and THZ1) barely suppress centromeric transcription, raising the possibility that unlike gene transcription, centromeric transcription might not require some essential transcription initiation factors.

Triptolide was previously used to study the function of centromeric transcription in *Drosophila* S2 cells (Bobkov et al., 2018). We therefore examined the extent to which it suppressed centromeric transcription in *Drosophila* S2 cells. Unlike in human cells, treatment of triptolide largely suppressed ongoing transcription on the centromere of the X-chromosome in S2 cells, as revealed by two pairs of centromere primers (Fig. S1 C; Usakin et al., 2007). These results suggest that centromeric DNA sequences might be a key factor for the efficacy of triptolide in the suppression of centromeric transcription, as they are very divergent across species (Kasinathan and Henikoff, 2018).

### Treatment of α-amanitin, not triptolide, induces severe centromeric cohesion defects in mitotic cells

**Figure 2.**
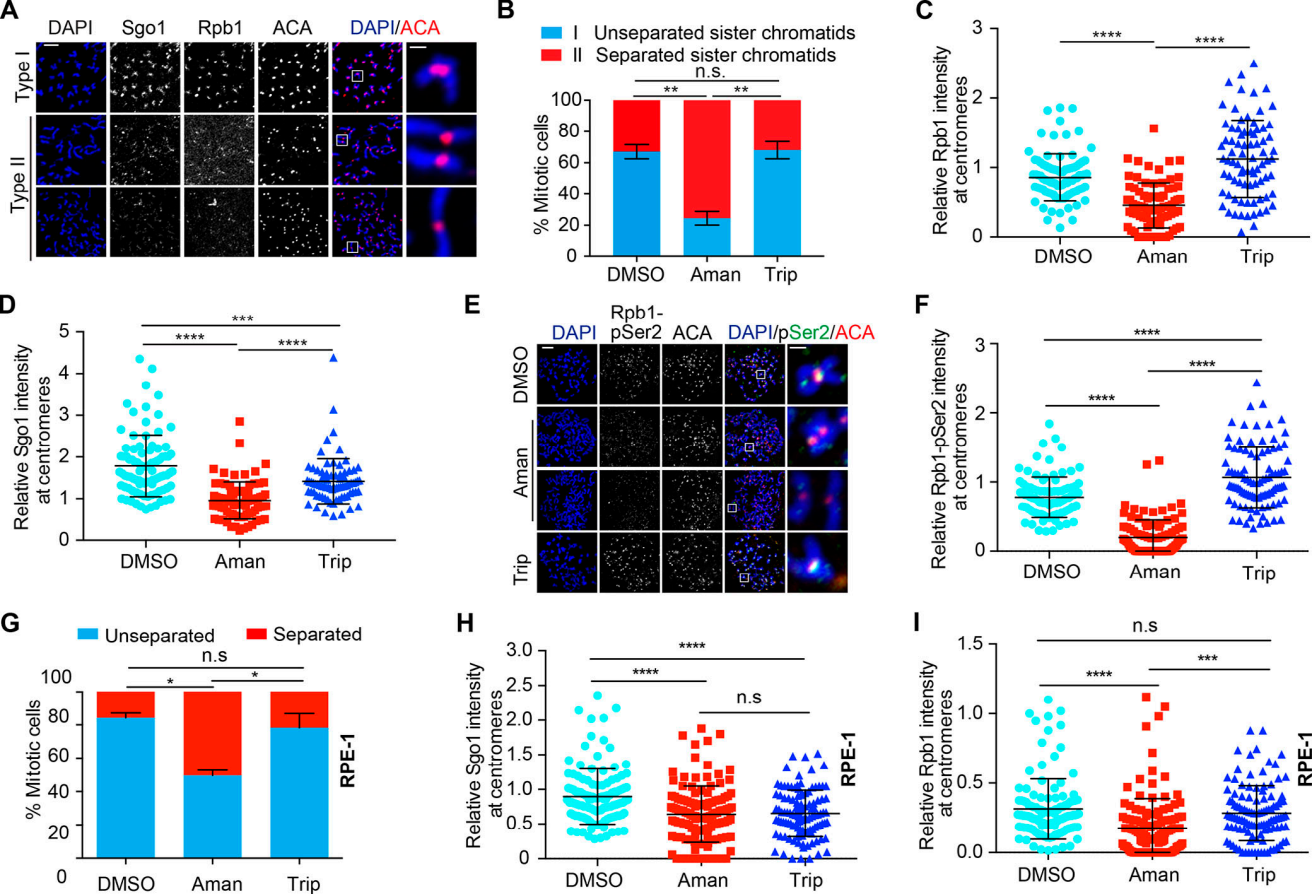
**α-amanitin, not triptolide, which suppresses ongoing centromeric transcription, impairs centromeric cohesion. (A)** Nocodazole-arrested HeLa Tet-On cells were treated with DMSO, α-amanitin (Aman), or triptolide (Trip) for 5 h and then subjected to chromosome spread and immunostaining with the indicated antibodies (Rbp1, 4H8). Two major types of chromosome morphology were observed: type I, chromosomes with cohesed sister centromeres and robust localization of Sgo1 and Rpb1 at centromeres, and type II, chromosomes with separated centromeres and decreased localization of Sgo1 and Rpb1 at centromeres. The scale bars in the left and right panels represent 5 µm and 1 µm, respectively. **(B)** Quantification of chromosome morphology with unseparated (type I) and separated sister chromatids (type II) in A. The average and standard error calculated from at least three independent experiments are shown here. At least 30 mitotic cells were analyzed for each condition in one single experiment. **(C** **and** **D)** Relative intensity of Rpb1 (Rpb1/ACA) and Sgo1 (Sgo1/ACA) at centromeres in A. Quantification details are recorded in Materials and methods. At least 90 centromeres (six per cell) were scored for each condition. The average and standard deviation are shown in C and D. **(E)** Nocodazole-arrested HeLa Tet-On cells were treated as described in A. Cells were incubated with antibodies against Rpb1-pSer2 (H5; BioLegend). The scale bars in the left and right panels represent 5 µm and 1 µm, respectively. **(F)** Relative intensity of Rpb1-pSer2 (pSer2/ACA) at centromeres in E. The experiment was repeated twice, and the results are highly reproducible. Quantification was performed based on the results from a single experiment. At least 90 centromeres (six per cell) were scored for each condition. The average and standard deviation are shown. **(G–I)** Nontransformed RPE-1 cells were treated under the same conditions as in A. Quantifications of chromosome morphology with unseparated and separated sister chromatids (G) and relative intensities of Rpb1 (I) and Sgo1 (H) at centromeres are shown here. The experiment was repeated at least three times. The average and standard error are shown in G. The average and standard deviation are shown in H and I. Quantifications for H and I****were performed based on a single experiment. At least 30 mitotic cells were analyzed for each condition in one single experiment (G). At least 90 centromeres (six per cell) were scored for each condition (H and I). *, P < 0.05; **, P < 0.01; ***, P < 0.001; ****, P < 0.0001.

With the above knowledge about these transcriptional inhibitors, we then tested how their application affected centromere functions in human cells. We focused on centromeric cohesion, as we had previously demonstrated that mitotic transcription promotes centromeric cohesion (Liu et al., 2015). Flavopiridol was not included here because it triggered a rapid escape from mitosis. Nocodazole-arrested HeLa Tet-On cells were treated with DMSO, α-amanitin, or triptolide, and then cells were subjected to chromosome spread for analysis of centromeric cohesion. We found that treatment of α-amanitin, which suppressed ongoing centromeric transcription, induced centromeric cohesion defects (Type II; Fig. 2 A) in ∼70% of cells, whereas DMSO treatment did so in ∼30% of cells (Fig. 2 B). Interestingly, treatment with triptolide, which poorly suppressed ongoing centromeric transcription, barely worsened centromeric cohesion (∼32%). These results may suggest that ongoing centromeric transcription is required for maintaining centromeric cohesion. Notably, cells with centromeric cohesion defects (Type II; Fig. 2 A) often exhibited reduced levels of Sgo1, total RNAPII (Rpb1, 4H8), and actively elongating RNAP II (Rpb1-pSer2, H5) at centromeres (Fig. 2, A and E). Quantification analysis indeed confirmed that the centromeric Sgo1, Rpb1, and Rpb1-pSer2 levels were significantly reduced in α-amanitin–treated cells compared with DMSO-treated cells, while they were either barely or slightly reduced in triptolide-treated cells (Fig. 2, C, D, and F). Similar results were also observed in nontransformed RPE-1 cells (Fig. 2, G–I). Thus, reduced centromeric transcription is likely responsible for impaired centromeric cohesion. The inability of triptolide to inhibit ongoing centromeric transcription and weaken centromeric cohesion may explain why no obvious mitotic progression defects were observed in triptolide-treated cells in a previous study (Novais-Cruz et al., 2018). In addition, treatment of α-amanitin did not significantly reduce the amounts of total centromeric RNAs but induced severe centromeric cohesion defects (Fig. 1 C and Fig. 2, B and G), suggesting that centromeric cohesion is maintained largely by actively ongoing centromeric transcription. Notably, treatment of triptolide did mildly decrease the total Sgo1 levels at centromeres but did not induce more severe centromeric cohesion defects. This is likely because the pool of Sgo1 that existed at inner-centromeres was sufficient to maintain centromeric cohesion, although total Sgo1 levels at centromeres were reduced (Liu et al., 2013a).

To address if α-amanitin–induced centromeric cohesion defects were due to reduced protein levels of cohesin subunits and cohesion regulators, we examined Smc1, Sororin, Sgo1, and Bub1 levels and found that none of them was decreased in cells treated with α-amanitin, triptolide, flavopiridol, or THZ1 (Fig. S2 B). In addition, treatment of α-amanitin and triptolide did not obviously change CENP-A levels at centromeres either (Fig. S2, C and D), suggesting that the observed phenotypes here are unlikely due to a significant change in CENP-A dynamics.

### Treatment of THZ1 strengthens centromeric cohesion

**Figure 3.**
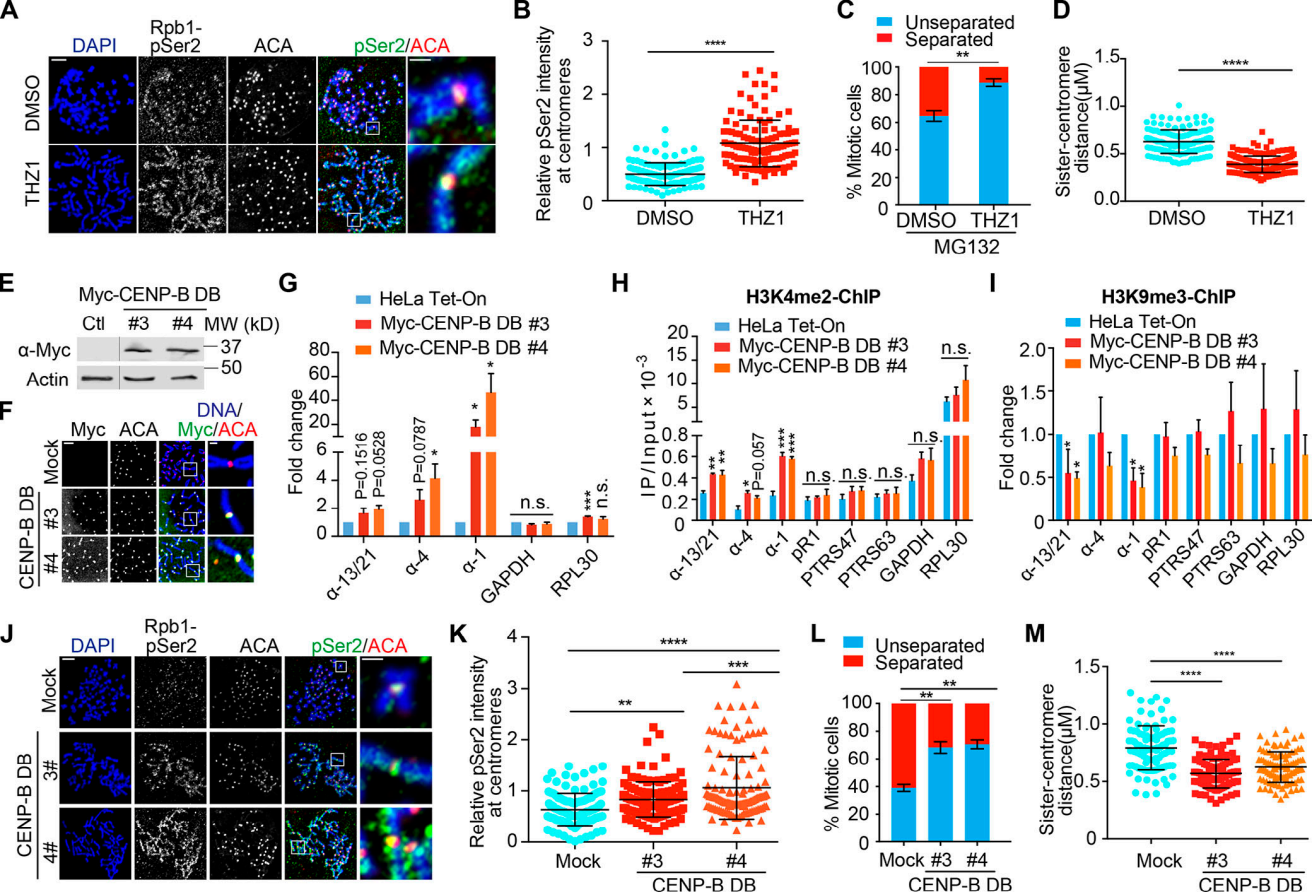
**THZ1 treatment and expression of CENP-B DB increase centromeric transcription and strengthen centromeric cohesion. (A)** Thymidine-arrested HeLa Tet-On cells were released into fresh medium containing DMSO or THZ1 and incubated for 12 h with the treatment of nocodazole in the last 2 h. Mitotic cells were collected and subjected for chromosome spread and immunostaining with the indicated antibodies. The scale bars in the left and right panels represent 5 µm and 1 µm, respectively. **(B)** Quantification of relative Rpb1-pSer2 intensity (pSer2/ACA) at centromeres in A. Quantification details are recorded in the Materials and methods. The experiment was repeated twice, and the results are highly reproducible. Quantification was performed based on the results from a single experiment. The average and standard deviation are shown here. At least 90 centromeres (six per cell) were scored for each condition. **(C)** Thymidine-arrested HeLa Tet-On cells were released into fresh medium containing DMSO or THZ1 and incubated for 12 h with the treatment of MG132 in the last 2 h. Mitotic cells were subjected to chromosome spread. Quantification of chromosome morphology with unseparated and separated sister chromatids is shown here. The average and standard error were calculated based on three independent experiments. At least 30 mitotic cells were scored for each condition in one single experiment. **(D)** Quantification of sister-centromere distance in cells with normal centromeric cohesion in C. The average and standard deviation are shown here. At least 150 centromeres (10 per cell) were scored for each condition. **(E)** Lysates of HeLa Tet-On cells (Ctl) and HeLa Myc–CENP-B DB stable cells treated with doxycycline were resolved with SDS-PAGE and blotted with the indicated antibodies. **(F)** Nocodazole-arrested HeLa Tet-On cells (Mock) and HeLa Myc–CENP-B DB stable cells treated with doxycycline were subjected to chromosome spread followed by staining with the indicated antibodies. The scale bars in the left and right panels represent 5 µm and 1 µm, respectively. **(G)** HeLa Tet-On cells and HeLa Myc–CENP-B DB stable cells were treated with doxycycline, and RNA was extracted and purified for real-time PCR analysis with the indicated primers. The average and standard error were calculated based on three independent experiments. **(H and I)** HeLa Tet-On cells and HeLa Myc–CENP-B DB stable cells treated with doxycycline were cross-linked with formaldehyde, sonicated, and then subjected to immunoprecipitation (IP) with H3K4me2 (H) and H3K9me3 (I) antibodies. DNA extracted from the immunoprecipitates was subjected to real-time PCR analysis with the indicated primers. The average and standard error were calculated based on three independent experiments. **(J)** Nocodazole-arrested HeLa Tet-On cells (Mock) and HeLa Myc–CENP-B DB stable cells treated with doxycycline were subjected to chromosome spread and staining with the indicated antibodies (Rpb1-pSer2, H5). The scale bars in the left and right panels represent 5 µm and 1 µm, respectively. **(K)** Quantification for relative intensity of Rpb1-pSer2 (pSer2/ACA) at centromeres in J. The experiment was repeated twice, and the results are highly reproducible. Quantification was performed based on the results from a single experiment. The average and standard deviation are shown here. At least 90 centromeres (six per cell) were scored for each condition. **(L)** HeLa Tet-On cells and Myc–CENP-B DB stable cells treated with doxycycline were incubated with MG132 for 2 h. Cells were then subjected to chromosome spread and staining with DAPI and ACA. Quantification for chromosome morphology with unseparated and separated sister chromatids is shown here. The average and standard error were calculated based on three independent experiments. At least 30 mitotic cells were scored for each condition in one single experiment. **(M)** Quantification for sister-centromere distance in cells with normal centromeric cohesion in L. Quantification was performed based on the results from a single experiment. Error bars represent mean and SD. At least 90 centromeres (six per cell) were scored for each condition. *, P < 0.05; **, P < 0.01; ***, P < 0.001; ****, P < 0.0001.

We next examined how treatment of THZ1, the inhibitor that significantly increased centromeric transcription, affected centromeric cohesion. Thymidine-arrested HeLa Tet-On cells were released into fresh medium containing THZ1 and incubated for 12 h, with nocodazole treatment in the last 2 h. Mitotic cells were collected and subjected to chromosome spread followed by immunostaining. We found that treatment of THZ1 induced increased Rpb1 and Rpb1-pSer2 levels at centromeres (Fig. 3, A and B; and Fig. S2, E and F), which may explain why centromeric transcription was enhanced in the previous experiments (Fig. 1 C). Strikingly, ectopic Rpb1, Rpb1-pSer2, and Sgo1 were also observed on chromosome arms in ∼50% of cells, whereas they were barely detected on chromosome arms in DMSO-treated cells under the same conditions (Fig. 3 A; and Fig. S2, E and F). These chromosomes with ectopic Rpb1 and Sgo1 always exhibited the morphology of arm close, which is likely caused by gain of cohesion. The phenotype of arm close is unlikely to be caused by decreased Wapl protein levels (Fig. S2 G).

To test if increased centromeric transcription could strengthen centromeric cohesion, we examined centromeric cohesion in the presence of MG132. Treatment of MG132 for 2 h induced centromeric cohesion defects (cohesion fatigue) in ∼36% of DMSO-treated HeLa Tet-On cells (Fig. 3 C; Daum et al., 2011). Interestingly, the defects were significantly alleviated in THZ1-treated cells (∼11%). Furthermore, by measuring the sister-centromere distance in cells exhibiting normal centromeric cohesion, we found the distance was shorter in THZ1-treated cells than in DMSO-treated cells (Fig. 3 D). All these results suggest that increased centromeric transcription strengthens centromeric cohesion. Notably, unlike THZ1, triptolide did not strengthen centromeric cohesion, although it also induced a mild increase in transcription of some, but not all, of the tested centromeres. As the extent of triptolide-increased centromeric transcription was lower than that triggered by THZ1 (Fig. 1 C), triptolide treatment may not be sufficient to systematically promote cohesion.

### Expression of CENP-B DB increases centromeric transcription and strengthens centromeric cohesion

The above findings strongly suggest that centromeric transcription promotes centromeric cohesion, but application of general transcription inhibitors still provoked the issue of whether the observed phenomena are a direct consequence of centromeric transcription suppression. To address this issue, we decided to develop a novel approach to specifically manipulate centromeric transcription without affecting the rest of the genome. Human α-satellite DNA repeats comprise a unique DNA element (B-box) that binds CENP-B (Masumoto et al., 2004), making it a potent carrier to target a transcriptional repressor to α-satellite DNA repeats at centromeres. As a matter of fact, the DB (1–163) of CENP-B has been applied to target proteins, specifically to centromeres (Zhang et al., 2020). Doxycycline-inducible HeLa Tet-On stable cells expressing Myc–CENP-B DB were constructed and validated by Western blots (Fig. 3 E). Immunostaining and chromatin immunoprecipitation (ChIP) experiments confirmed that Myc–CENP-B DB was specifically targeted to centromeres (Fig. 3 F and Fig. S2 H). We then examined if expression of Myc–CENP-B DB per se would alter centromeric transcription by measuring the amounts of total centromeric RNAs. Myc–CENP-B DB stable cells (#3 and #4) were incubated with doxycycline for 48 h, and total RNAs were isolated for real-time PCR analysis using gene primers GAPDH and PRL30 and centromere primers α-1, α-4, and α-13/21. Surprisingly, we found that targeting of Myc–CENP-B DB to centromeres overall enhanced transcription at centromeres without significantly affecting gene transcription (Fig. 3 G). Especially, transcription on α-1 DNAs was increased by more than 20-fold.

**Figure S3.**
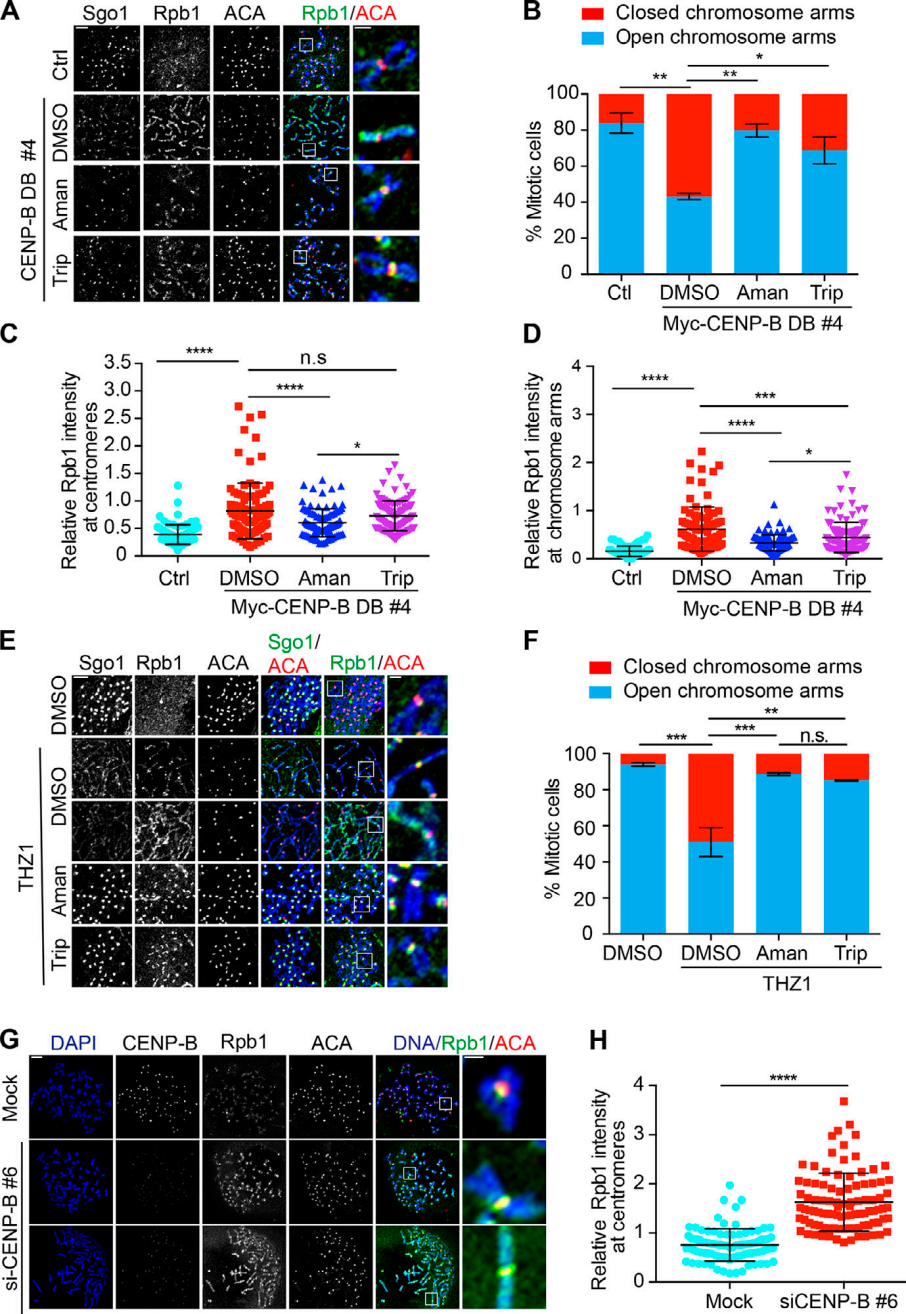
**Induced cohesion on chromosome arms depends on transcription. (A)** Thymidine-arrested HeLa Tet-On cells (Ctrl) and HeLa Myc–CENP-B DB stable cells treated with doxycycline were released into fresh medium and further incubated for 12 h. At 8 h after release, DMSO, α-amanitin (Aman), and triptolide (Trip) were added. 2 h before harvest, cells were treated with nocodazole. Collected mitotic cells were subjected to chromosome spread and staining with the indicated antibodies. The scale bars in the left and right panels represent 5 µm and 1 µm, respectively. **(B)** Quantification for chromosome morphology with closed and open chromosome arms is shown here. The average and standard error were calculated based on three independent experiments. At least 30 mitotic cells were scored for each condition in one single experiment. **(C** **and** **D)** Relative Rpb1 (4H8) intensity at centromeres (C) and on chromosome arms (D) is shown here. The quantification details are recorded in Materials and methods. The average and standard deviation are shown. At least 90 centromeres (six per cell; H) and 90 chromosome arms (six per cell; I) were scored for each condition in one single experiment. **(E)** Thymidine-arrested HeLa Tet-On cells were released into fresh medium containing DMSO or THZ1 and incubated for 12 h with the treatment with nocodazole in the last 2 h. Cells were further treated with DMSO, α-amanitin (Aman), or triptolide (Trip) for 4 h before harvest. Collected mitotic cells were subjected to chromosome spread and immunostaining with the indicated antibodies. The scale bars in the left and right panels represent 5 µm and 1 µm, respectively. **(F)** Quantification for cells in E with open and closed chromosome arms. The average and standard error were calculated based on three independent experiments. At least 90 chromosome arms (six per cell; I) were scored for each condition in one single experiment. **(G)** HeLa Tet-On cells with mock treatment or CENP-B siRNA treatment were incubated with nocodazole for 2 h. Mitotic cells were subjected to chromosome spread and staining with DAPI and the indicated antibodies. The scale bars in the left and right panels represent 5 µm and 1 µm, respectively. **(H)** Quantification of relative Rpb1 (Rpb1/ACA) intensity in G at centromeres. The experiment was repeated twice, and the results are highly reproducible. Quantification was performed based on the results from a single experiment. The average and standard deviation are shown here. At least 90 centromeres (six per cell) were scored for each condition. *, P < 0.05; **, P < 0.01; ***, P < 0.001; ****, P < 0.0001. Mock, luciferase siRNAs.

To further understand the mechanism underlying Myc–CENP-B DB-enhanced centromeric transcription, we examined the H3K4 di-methylation (me2) levels at centromeres, as this has been shown to be an important epigenetic mark associated with active transcription at centromeres (Bergmann et al., 2011; Sullivan and Karpen, 2004). Myc–CENP-B DB stable cells (#3 and #4) were incubated with doxycycline for 48 h, and ChIP experiments were implemented followed by real-time PCR analysis using two pairs of gene primers (GAPDH and PRL30), three pairs of centromere primers (α-1, α-4, and α-13/21), and three pairs of pericentromere primers (PR1, PTRS47 and PTRS63; Jarmuż et al., 2007). We found that the H3K4me2 levels were increased at the tested centromeres but not at the tested pericentromeres and gene regions (Fig. 3 H). At the same time, expression of Myc–CENP-B DB decreased the levels of the heterochromatin mark H3K9me3 at centromeres (Fig. 3 I). Thus, Myc–CENP-B DB-enhanced centromeric transcription is likely attributed to the formation of more transcription-permissible chromatin, but the exact underlying mechanism still remains unknown. Consistently, expression of Myc–CENP-B DB also increased the Rpb1 and Rpb1-pSer2 levels at centromeres (Fig. 3, J and K; and Fig. S3, A and C).

As THZ1-induced centromeric transcription strengthens centromeric cohesion, we wanted to know if Myc–CENP-B DB-enhanced centromeric transcription could do so as well. Myc–CENP-B DB stable cells were incubated with doxycycline for 48 h and treated with MG132 in the last 2 h. Mitotic cells were collected for chromosome spread. We found that ∼60% of control cells exhibited centromeric cohesion defects (Fig. 3 L). Expression of Myc–CENP-B DB in the two stable cell lines significantly alleviated centromeric cohesion defects (∼30%) and also shortened the sister-centromere distance in cells exhibiting normal centromeric cohesion (Fig. 3, L and M). These results further confirm that ectopically increased centromeric transcription strengthens centromeric cohesion. Expression of CENP-B DB might alter CENP-A dynamics, thus affecting centromeric cohesion (Fachinetti et al., 2015). We therefore examined the CENP-A levels at centromeres in cells expressing CENP-B DB, and no significant change was observed (Fig. S2 I), suggesting that CENP-B DB-associated phenotypes might not be due to a significant change in CENP-A dynamics. Notably, while our findings clearly demonstrated that increased centromeric transcription by THZ1 and CENP-B DB enhances centromeric cohesion, the exact underlying mechanisms are unknown. It is also possible that these observed phenotypes might not be a direct consequence of altered centromeric transcription, as THZ1 treatment and CENP-B DB expression could directly affect cohesin dynamics in interphase.

### CENP-B DB-induced chromosome arm close depends on transcription

Expression of Myc–CENP-B DB also induced chromosome arm close. We then sought to determine if this phenotype was dependent on transcription. Myc–CENP-B DB stable cells (#4) treated with doxycycline were arrested with thymidine, then released into fresh medium and further incubated for 12 h with nocodazole treatment in the last 2 h. At 4 h before harvest, cells were also treated with DMSO, α-amanitin, or triptolide. We have shown that treatment of these two inhibitors for 4 h was able to suppress gene transcription but not centromeric transcription (Fig. 1 C), which allowed us to specifically assess the dependency of chromosome arm cohesion on transcription. Consistently, the Rpb1 levels on both centromeres and chromosome arms were significantly increased in the stable cells compared with control cells (Fig. S3, A and D). Treatment of α-amanitin or triptolide for 4 h either moderately or barely decreased centromeric Rpb1 levels in stable cells (Fig. S3, A and C), whereas treatment of α-amanitin or triptolide not only completely eliminated CENP-B DB-induced chromosome arm close (Fig. S3, A and B) but also significantly reduced Rpb1 levels on chromosome arms (Fig. S3, A and D). These results suggest that transcription on chromosome arms also promotes sister chromatid cohesion during mitosis.

Targeting of Myc–CENP-B DB to centromeres could impair the localization of Bub1 kinase to kinetochores, which in turn leads to chromosome arm close and ectopic accumulation of Sgo1 on chromosome arms (Liu et al., 2013a). We thereby examined the Bub1 levels at kinetochores in Myc–CENP-B DB stable cells and found that they were barely altered (Fig. S2, J and K), thereby excluding the possibility of Bub1 involvement in the Myc–CENP-B DB-induced phenotypes. In addition, we also found that THZ1-induced chromosome arm close also depends on transcription (Fig. S3, E and F). However, it is worth mentioning that the phenotype of chromosome arm close might also be caused by ectopic recruitment of Sgo1 onto chromosome arms by the retained RNAPII, as Sgo1 physically interacts with RNAPII (Liu et al., 2015).

### CENP-B knockdown increases centromeric transcription and strengthens centromeric cohesion

**Figure 4.**
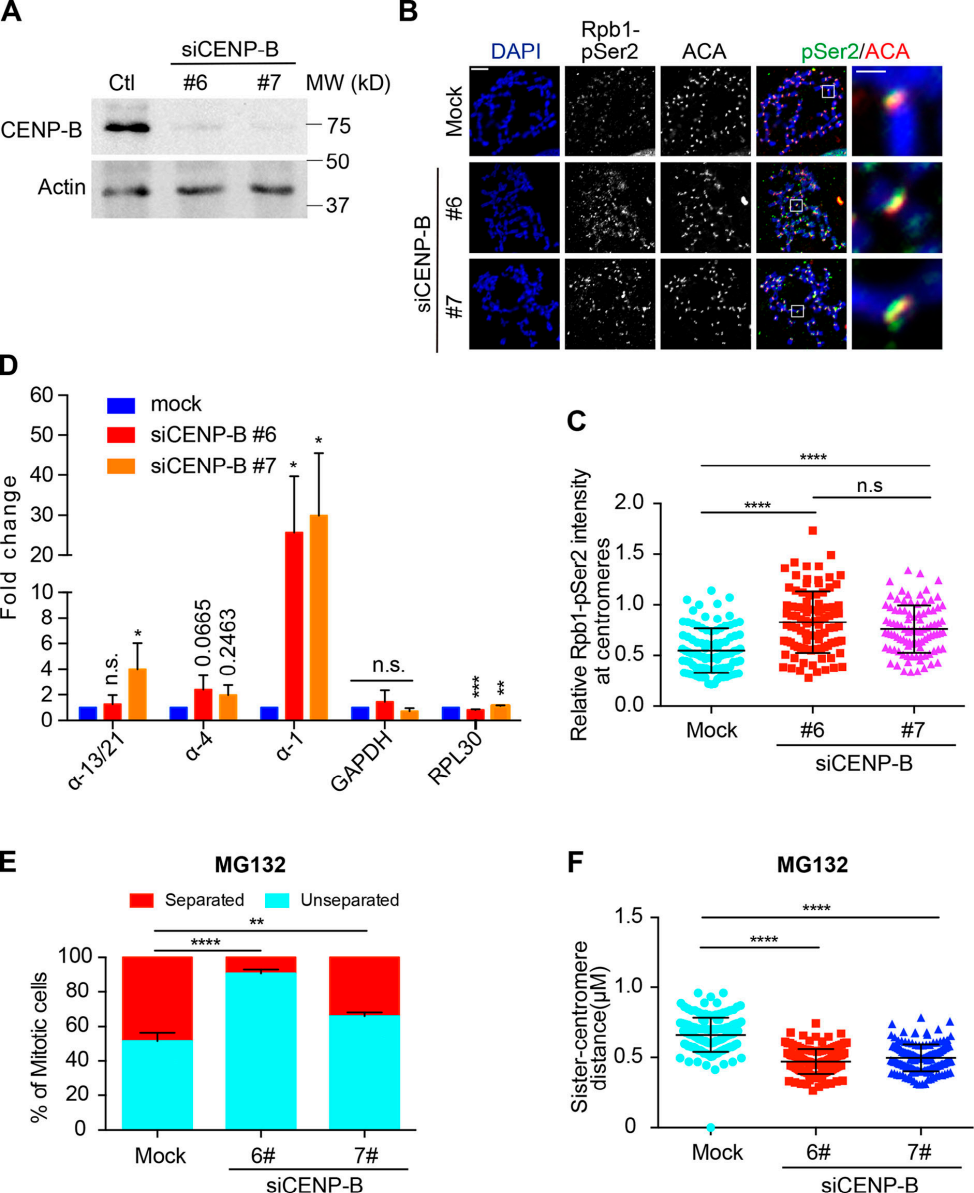
**CENP-B knockdown increases centromeric transcription and strengthens centromeric cohesion. (A)** Lysates of HeLa Tet-On cells with mock treatment or treated with CENP-B siRNAs were resolved with SDS-PAGE and blotted with the indicated antibodies. **(B)** HeLa Tet-On cells with mock treatment or treated with CENP-B siRNAs were incubated with nocodazole for 2 h. Mitotic cells were subjected to chromosome spread followed by staining with DAPI and the indicated antibodies. The scale bars in the left and right panels represent 5 µm and 1 µm, respectively. **(C)** Quantification for relative intensity of Rpb1-pSer2 (pSer2/ACA) at centromeres in B. Quantification details are recorded in the Materials and methods. The experiment was repeated three times, and the results are highly reproducible. Quantification was performed based on the results from a single experiment. The average and standard deviation are shown here. At least 90 centromeres (six per cell) were scored for each condition. **(D)** RNA extracted from HeLa Tet-On cells with mock treatment or treated with CENP-B siRNAs was subjected to real-time PCR analysis with the indicated primers. The average and standard error were calculated based on three independent experiments. **(E)** HeLa Tet-On cells and Myc–CENP-B DB stable cells treated with doxycycline were incubated with MG132 for 2 h. Cells were then subjected to chromosome spread and staining with DAPI and ACA. Quantification for chromosome morphology with unseparated and separated sister chromatids is shown here. The average and standard error were calculated based on three independent experiments. At least 30 mitotic cells were scored for each condition in one single experiment. **(F)** Quantification for sister-centromere distance in cells with normal centromeric cohesion in E. Quantification was performed based on the results from a single experiment. The average and standard deviation are shown here. At least 150 centromeres (10 per cell) were scored for each condition. *, P < 0.05; **, P < 0.01; ***, P < 0.001; ****, P < 0.0001. Ctl, control; Mock, luciferase siRNAs.

CENP-B DB-induced centromeric transcription suggests that CENP-B might function as a suppressor for centromeric transcription. To test it, we examined centromeric RNAPII levels by immunostaining and centromeric transcription by real-time PCR in CENP-B knockdown cells. Western blots and immunostaining experiments demonstrated that the protein levels of CENP-B were significantly decreased by CENP-B siRNAs (Fig. 4 A and Fig. S3 G). Similar to CENP-B DB expression, CENP-B knockdown significantly increased Rpb1 and Rpb1-pSer2 levels at centromeres (Fig. 4, B and C; and Fig. S3, G and H). Accordingly, the amounts of the tested centromeric transcripts were also increased (Fig. 4 D), which is consistent with a recent report showing that CENP-B knockout increased centromeric RNA Fluorescence In Situ Hybridization signals (Bury et al., 2020). We next examined if CENP-B knockdown could strengthen centromeric cohesion. Similar to CENP-B DB expression, CENP-B knockdown significantly suppressed the centromeric cohesion defects caused by MG132 and also decreased the sister-centromere distance in cells exhibiting normal centromeric cohesion (Fig. 4, E and F). Taking these results together, we make a conclusion that centromeric transcription promotes centromeric cohesion.

### Targeting of the transcriptional suppressor Kox-1 with CENP-B DB to centromeres decreases centromeric transcription and impairs centromeric cohesion

**Figure 5.**
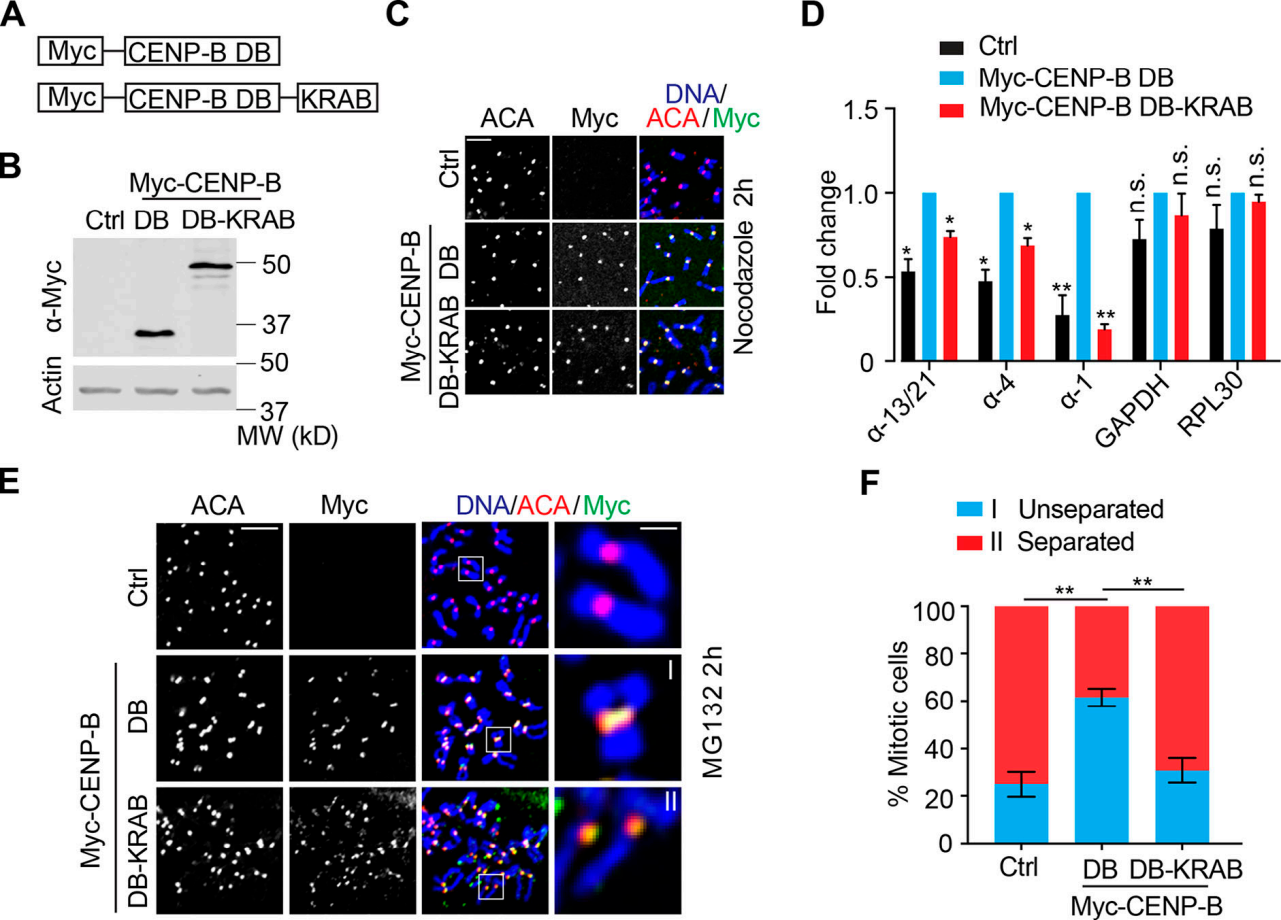
**Ectopic targeting of the KRAB of Kox-1 to centromeres decreases centromeric transcription and impairs centromeric cohesion. (A)** Schematic drawing of CENP-B DB-KRAB fusion proteins. **(B–D)** HeLa Tet-On cells transfected with vectors (Ctrl) or plasmids containing Myc–CENP-B DB or DB-KRAB were subjected to Western blots (B), immunostaining (C), or real-time analysis for RNA (D). The average and standard error in D were calculated from three independent experiments. The scale bar in C represents 5 µm. **(E)** HeLa Tet-On cells with mock transfection (Ctrl) or transfected with plasmids containing Myc–CENP-B DB or DB-KRAB were treated with MG132 for 2 h, and mitotic cells were subjected to chromosome spread and stained with the indicated antibodies. The scale bars in the left and right panels represent 5 µm and 1 µm, respectively. **(F)** Quantification for chromosome morphology with unseparated and separated sister chromatids in E. The average and standard error were calculated based on three independent experiments. At least 30 mitotic cells were scored for each condition in one single experiment. *, P < 0.05; **, P < 0.01.

We next tested if specific targeting of a transcriptional suppressor to centromeres with Myc–CENP-B DB could suppress centromeric transcription. The transcriptional suppressor Kox1 was selected, as it has been extensively used in CRISPR interference to suppress gene transcription (Gilbert et al., 2013). Therefore, we constructed a fusion protein (Myc–CENP-B DB-KRAB) that contains Myc–CENP-B DB and the KRAB domain (1–90) of Kox1 (Fig. 5, A and B). HeLa Tet-On cells transiently expressing Myc–CENP-B DB or Myc–CENP-B DB-KRAB were subjected to immunostaining, which demonstrated that both Myc–CENP-B DB and Myc–CENP-B DB-KRAB were localized well to centromeres (Fig. 5 C). We then tested if this approach could specifically suppress transcription at centromeres. HeLa Tet-On cells were transiently transfected with plasmids containing Myc–CENP-B DB or Myc–CENP-B DB-KRAB, and total RNAs were extracted for real-time PCR analysis. Consistently, transient expression of Myc–CENP-B DB significantly increased the expression of all the tested α-satellite RNAs, whereas transient expression of Myc–CENP-B DB-KRAB decreased them to levels comparable to those of control cells. Expression of Myc–CENP-B DB and Myc–CENP-B DB-KRAB barely altered the expression of GAPDH and RPL30 RNAs (Fig. 5 D). Thus, targeting the KRAB domain to centromeres using CENP-B DB is an effective way to specifically suppress centromeric transcription.

We then tested if this could weaken centromeric cohesion. HeLa Tet-On cells transiently transfected with plasmids containing Myc–CENP-B DB or Myc–CENP-B DB-KRAB were treated with MG132 for 2 h and then subjected to chromosome spread. Consistently, expression of Myc–CENP-B DB largely suppressed the centromeric cohesion defects induced by MG132 in control cells, whereas expression of Myc–CENP-B DB-KRAB failed to do so (Fig. 5, E and F). Notably, the fusion protein CENP-B DB-KRAB did not decrease centromeric transcription to levels lower than the ones in control cells. This could simply be due to insufficient expression of the fusion protein. Alternatively, this fusion protein might act in a dominant-negative manner. Nevertheless, the robustness of centromeric cohesion well echoes the overall changes in centromeric transcription by CENP-B DB and CENP-B DB-KRAB expression, suggesting that specifically decreasing centromeric transcription can weaken centromeric cohesion.

In summary, using transcriptional inhibitors, CENP-B DB, and CENP-B knockdown, we have manipulated centromeric transcription and, based on this, revealed that a major function of centromeric transcription in human cells is to maintain centromeric cohesion, which is essential for proper chromosome segregation. As treatment of transcriptional inhibitors for a few hours dramatically decreased the ongoing centromeric transcription but seemed not to do so for total RNAs, we believe that actively ongoing transcription rather than RNA transcripts plays a more important role in maintaining centromeric cohesion. This might explain why RPE-1 cells still exhibit strong centromeric cohesion even though they have a low amount of total centromeric RNA transcripts (Bury et al., 2020). Our findings here also suggest that increased centromeric transcription overall strengthens centromeric cohesion. However, it is not clear whether there exists a critical threshold for centromeric transcription: when it is surpassed, the positive role of centromeric transcription in centromeric cohesion would move in the opposite direction. Mechanistically, during mitosis, RNAPII transcriptional machinery may drive the critical cohesion protector Sgo1 to inner centromeres where it binds centromeric cohesin and protects cohesion (Liu, 2016; Liu et al., 2015). Notably, a recent study indicated that cohesin per se can also be a promoting factor for mitotic transcription (Perea-Resa et al., 2020). Thus, taking all the findings together, cohesin, transcription, and Sgo1 may form a positive feedback loop to regulate centromeric cohesion during mitosis (Zhang and Liu, 2020). Our findings suggest that this feedback loop may also exist on chromosome arms to regulate sister chromatid cohesion there. Finally, differential behaviors of transcriptional inhibitors on the transcription of genes and centromeres may suggest that the regulation of centromeric transcription is distinct from the regulation of canonical gene transcription. The non–B type DNA structures that centromeres form in many species might allow centromeric transcription to be less dependent on some transcriptional initiation factors (Kasinathan and Henikoff, 2018). In the future, understanding how centromeric transcription is regulated will be important in the field.

## Materials and methods

### Mammalian cell culture, transfection, and transcriptional inhibitors

HeLa Tet-On cells (Invitrogen) were cultured in DMEM (Invitrogen) supplemented with 10% fetal bovine serum and 10 mM L-glutamine at 37°C and 5% CO_2_. RPE-1 cells (a gift from Dr. Hongtao Yu, University of Texas Southwestern Medical Center at Dallas, Dallas, TX) were incubated in DMEM: F-12 medium (Invitrogen) supplemented with 10% fetal bovine serum and 10 mM L-glutamine at 37°C and 5% CO_2_. *Drosophila* S2 cells were cultured in Schneider’s Medium (Invitrogen) at room temperature (23°C) supplemented with 10% fetal bovine serum. Nocodazole (M1404) and MG132 (474790) were purchased from Sigma-Aldrich.

Plasmid transfection was performed with the Effectene reagent (Qiagen; 301425) according to the manufacturer’s protocols. To generate the inducible stable cell lines, HeLa Tet-On cells were transfected with pTRE2 vectors encoding MYC–CENP-B DB and selected with 350 μg ml^−1^ hygromycin (Invitrogen; 10687010). Expression of the desired proteins in the surviving clones was screened in the presence of 1 µg ml^−1^ doxycycline (Invitrogen; D9891).

The following transcriptional inhibitors were used in this study: α-amanitin (MilliporeSigma; A2263), flavopiridol (Selleckchem; S1230), triptolide (MilliporeSigma; T3652), and THZ1 (Selleckchem; S7549). These inhibitors were dissolved in DMSO, and working concentrations were specified in each experiment.

For RNAi experiments, siRNA oligonucleotides were purchased from Dharmacon. HeLa cells were transfected using Lipofectamine RNAiMax (Invitrogen) and analyzed at 48–72 h after transfection. The sequences of the siRNAs used in this study: siLuciferase, 5'-UCA​UUC​CGG​AUA​CUG​CGA​UUU-3'; siCENP-B #6, 5′-GCA​CGA​UCC​UGA​AGA​ACA​A-3′ (Dharmacon; D-003250-06); siCENP-B #7, 5′-GGA​GGA​GGG​UGA​UGU​UGA​U-3′ (Dharmacon; D-003250-07); and siWapl, 5′-CGG​ACT​ACC​CTT​AGC​ACA​A-3′.

### Antibodies and immunoblotting

The following antibodies were used in this study: anti-centromere antibody (ACA; or CREST-ImmunoVision; HCT-0100), anti-Myc (Roche; 11667203001), anti-Smc1 (Bethyl; A300-055A), anti-Rpb1 (Abcam; ab5408), anti-H3K4me2 (EMD Millipore; 07–030), anti-H3K9me3 (Abcam; ab8898), anti-Actin (Invitrogen; MA5-11869), anti-CENP-B (Abcam; ab25734), anti–CENP-A (EMD Millipore; 07–574), and anti–Rpb2-pSer2 (BioLegend; H5). Anti-Sgo1, anti-Bub1, and anti-Wapl were made in-house as described previously (Liu et al., 2013a; Qu et al., 2019). Anti-Sororin antibodies described previously were a gift from Dr. Susannah Rankin at Oklahoma Medical Research Foundation, Oklahoma City, OK (Liu et al., 2013b).

The secondary antibodies were purchased from LI-COR: Goat anti-Mouse IgG Secondary Antibody (926–68070) and Goat anti-Rabbit IgG Secondary Antibody (926–32211).

For immunoblotting, primary and secondary antibodies were used at 1-µg ml^−1^ concentration.

### EU chasing and purification of EU-RNAs

Purification of EU-RNAs was performed according to the protocol from Click-iT Nascent RNA Capture Kit (Thermo Fisher Scientific; C10365). Cells with a confluency of 60–80% in 10-cm petri dishes were treated with EU in a final concentration of 0.5 mM for 1 h. EU-chased cells were then collected and dissolved in TRIzol solution (Invitrogen; 15596026). Total RNA was extracted, dissolved in nuclease-free water, and treated with TURBO DNase (Invitrogen; AM2238) in the presence of RNase inhibitor (NEB; M3014) at 37°C for 45 min. Total RNA was then extracted with Phenol/Chloroform/Isoamyl alcohol (25:24:1, vol/vol; Invitrogen; 15593–031), precipitated with ice-cold ethanol solution containing glycogen and sodium acetate, and finally dissolved in nuclease-free water. Purified total RNA was then further incubated with streptavidin dynabeads pretreated with Salmon Sperm DNA (Invitrogen; 15632–011) in binding buffer for 45 min. With the help of DynaMag-2 Magnet (Invitrogen; 12321D), dynabeads were washed with wash buffer I and II.

### Reverse transcription and real-time PCR analysis

EU-RNA–bound dynabeads were mixed with iScript Reverse Transcription Supermix (Bio-Rad; 1708841), and reverse transcription was performed according to the manufacturer’s protocols. After being mixed with the SsoAdvanced Universal SYBR Green Supermix (Bio-Rad; 1725274), the synthesized cDNA was subjected to real-time PCR analysis using QuantStudio 6 Flex Real-Time PCR System (Applied Biosystems).

The following primers for human cells were used in this study: GAPDH-F: 5′-TGA​TGA​CAT​CAA​GAA​GGT​GGT​GAA​G-3′, GAPDH-R: 5′-TCC​TTG​GAG​GCC​ATG​TGG​GCC​AT-3′; Rpl30-F: 5′-CAA​GGC​AAA​GCG​AAA​TTG​GT-3′, Rpl30-R: 5′-GCC​CGT​TCA​GTC​TCT​TCG​ATT-3′; SAT-1-F: 5′-AAG​GTC​AAT​GGC​AGA​AAA​GAA-3′, SAT-1-R: 5′-CAA​CGA​AGG​CCA​CAA​GAT​GTC-3′; SAT-4-F: 5′-CAT​TCT​CAG​AAA​CTT​CTT​TGT​GAT​GTG-3′, SAT-4-R: 5′-CTT​CTG​TCT​AGT​TTT​TAT​GTG​AAT​ATA-3′; SAT13/21-F: 5′-TAG​ACA​GAA​GCA​TTC​TCA​GAA​ACT-3′, SAT-13/21-R: 5′-TCC​CGC​TTC​CAA​CGA​AAT​CCT​CCA​AAC-3′; pTRS-63-F: 5′-ATT​GAA​ACC​TGC​TCG​ATT​GG-3′, pTRS-63-R: 5′-TCG​GTT​TGA​TTC​CAT​TCC​AT-3′; pR1-F: 5′-CTG​GAC​TTT​GGT​GGA​AAG​GA-3′, pR1-R: 5′-ACA​ATC​TCA​GCC​CAC​ATT​CC-3′; pTRS-47-F: 5′-GGA​TCA​GAA​CGG​AAC​AGA​GC-3′, PTRS-47-R: 5′-AGT​CCA​CTG​CAT​TCC​ATT​CC-3′; C-Myc-1-F: 5′-CCT​GGT​GCT​CCA​TGA​GGA​GAC-3′, C-Myc-1-R: 5′-CAG​ACT​CTG​ACC​TTT​TGC​CAG​G-3′; C-Myc-2-F: 5′-AAA​CAC​AAA​CTT​GAA​CAG​CTA​C-3′, C-Myc-2-R: 5′-ATT​TGA​GGC​AGT​TTA​CAT​TAT​GG-3′.

The primers for *Drosophila* S2 cells used in this study were described previously (Usakin et al., 2007): CEN361-R (right): 5′-TCA​ACG​ATG​TAT​GAC​ATT​CC-3′, CEN361-L (left): 5′-TGA​GCT​CGT​AAT​AAA​ATT​TCC-3′; CEN359-R: 5′-TAT​TCT​TAC​ATC​TAT​GTG​ACC-3′, CEN359-L: 5′-GTT​TTG​AGC​AGC​TAA​TTA​CC-3′; Rp49: 5′-ATG​ACC​ATC​CGC​CCA​GCA​TAC-3′ and 5′-CTG​CAT​GAG​CAG​GAC​CTC​CAG-3′; and Pgd: 5′-AGG​ACT​CGT​GGC​GCG​AGG​TG-3′ and 5′-GGA​ATG​TGT​GAA​CGG​GAA​AGT​GGA​G-3′.

### ChIP

Nocodazole-arrested mitotic cells were first cross-linked with buffer (50 mM Hepes, pH 8.0, 1% formaldehyde, 100 mM NaCl, 1 mM EDTA, and 0.5 mM EGTA) at room temperature for 10 min and further treated with 125 mM glycine for another 5 min. Cells were then resuspended in immunoprecipitation buffer (10 mM Tris, 300 mM NaCl, 1 mM EDTA, 1 mM EGTA, 1% Triton X-100, and 1% sodium deoxycholate) and sonicated using a Thermo Fisher Scientific sonicator. After centrifugation, the supernatant of cell lysates was precleared with protein-A beads (Santa Cruz; SC-2001) at 4°C for 2 h. Precleared cell lysates were incubated with 5 µg anti-H3K4me2 antibodies overnight and further with protein-A beads for another 2 h at 4°C. Pelleted beads were sequentially washed by low salt buffer (20 mM Tris 8.0, 150 mM NaCl, 0.1% SDS, 1% Triton X-100, and 2 mM EDTA), high-salt buffer (20 mM Tris 8.0, 500 mM NaCl, 0.1% SDS, 1% Triton X-100, and 2 mM EDTA), LiCl buffer (10 mM Tris 8.0, 0.25 M LiCl, 1% IGEPAL CA630, 1% sodium deoxycholate, and 1 mM EDTA), and TE buffer (10 mM Tris, pH 8.0, and 1 mM EDTA, pH 8.0). After the last TE buffer wash, beads were treated with elution buffer (10 mM Tris 8.0, 1 mM EDTA, and 1% SDS) at 65°C for 10 min, and the supernatant was further incubated at 65°C overnight to reverse the cross-linking. Then the solution was sequentially treated with RNase A (Qiagen; 1007885) at 37°C for 1 h and Proteinase K (Thermo Fisher Scientific; EO0491) at 50°C for 2 h. Finally, DNA in the solution was extracted with Phenol/Chloroform/Isoamyl alcohol (25:24:1, vol/vol; Invitrogen; 15593–031) and purified by Qiagen gel purification kit for real-time PCR analysis.

### Immunofluorescence and chromosome spread

Chromosome spread and immunostaining were performed as described before (Liu et al., 2013a). Nocodazole or MG132-arrested mitotic cells were swelled in a prewarmed hypotonic solution containing 50 mM KCl for 15 min at 37°C and then spun onto slides with a Shandon Cytospin centrifuge. Cells were treated with ice-cold PBS containing 0.2% Triton X-100 for 2 min and then with 4% ice-cold paraformaldehyde for 4 min. After being washed with PBS, cells were incubated with primary antibodies overnight at 4°C. Cells were then washed with PBS containing 0.1% Triton X-100 and treated at room temperature for 1 h with the appropriate secondary antibodies conjugated to fluorophores (Invitrogen; A11008, A21090, and A31571). After incubation, cells were washed again with PBS containing 0.1% Triton X-100, stained with 1 µg ml^−1^ DAPI, and mounted with Vectashield. The images were taken by a Nikon inverted confocal microscope (Eclipse Ti2; NIS-Elements software) with a ×60 objective. Image processing was performed with ImageJ and Adobe Photoshop. Quantification was performed with ImageJ. Statistical analysis was performed with GraphPad Prism.

### Quantification and statistical analysis

Numeric intensities of experimental subjects under investigation were obtained with ImageJ. In the experiments of Fig. 2, C, D, F, H, and I; Fig. 3, B and K; Fig. 4 C; Fig. S2, D, F, I, and K; and Fig. S3, C, D, and H, six kinetochores were randomly selected from each cell. A mask was generated to mark centromeres on the basis of ACA fluorescence signals in the projected image. After background subtraction, the intensities of Sgo1, Rpb1, and Rpb1-pSer2 and ACA signals within the mask were obtained in number. Relative intensity was calculated from the intensity of Sgo1, Rpb1, or Rpb1-pSer2 signals normalized to that of ACA signals and plotted with the GraphPad Prism software. All experiments were repeated at least two times.

For quantification of CENP-A levels in Fig. S2, D and I, a mask was generated to mark centromeres on the basis of ACA fluorescence signals in the projected image. After background subtraction, the intensities of CENP-A and DNA signals within the mask were obtained in number. Relative intensity was calculated from the intensity of CENP-A signals normalized to that of DNA signals and plotted with GraphPad Prism software. All the experiments were repeated at least two times.

For quantification of Bub1 levels in Fig. S2 K, a mask was generated to mark centromeres on the basis of ACA fluorescence signals in the projected image. After background subtraction, the intensities of Bub1 and ACA signals within the mask were obtained in number. Relative intensity was calculated from the intensity of Bub1 signals normalized to that of ACA signals and plotted with GraphPad Prism software. All the experiments were repeated at least two times.

For quantification of Rpb1 levels on chromosome arms in Fig. S3 D, a mask was generated to mark a chromosome arm. After background subtraction, the intensities of Rpb1 and DAPI fluorescence signals within the mask were obtained in number. Relative intensity was derived from the intensity of Rpb1 normalized to that of DAPI signals and plotted with the GraphPad Prism software.

Measurement of sister-centromere distance in Fig. 3, D and M, and Fig. 4 F was performed with ImageJ. A straight line was drawn between a pair of sister centromeres, revealed by ACA signals. Numeric values were automatically generated by ImageJ.

Quantification was usually performed based on the results from a single experiment or three independent experiments, as specified in the legends of each experiment. Differences were assessed using ANOVA, followed by pairwise comparisons using Tukey’s test. All the samples analyzed were included in quantification. Sample size is recorded in the figures and their corresponding legends. No specific statistical methods were used to estimate sample size. No methods were used to determine whether the data met the assumptions of the statistical approach.

### Online supplemental material

Fig. S1 shows the validation of primers used in this study and the efficacies of various transcriptional inhibitors on the suppression of ongoing α-satellite and gene transcription. Fig. S2 demonstrates CENP-A levels at centromeres in cells treated with transcriptional inhibitors and stably expressing Myc–CENP-B DB, Bub1 levels at centromeres in cells stably expressing Myc–CENP-B DB, the relative expression level of C-MYC genes in cells treated with THZ1, protein levels of various cohesion-related regulators in cells treated with transcriptional inhibitors, and Rpb1 levels at centromeres in cells treated with THZ1. Fig. S3 includes the morphology of chromosome arms and Rpb1 levels on chromosomes in stable cells expressing Myc–CENP-B DB treated with α-amanitin or triptolide, the morphology of chromosome arms in THZ1-treated cells further incubated with α-amanitin or triptolide, and Rpb1 levels at centromeres in cells treated with CENP-B siRNAs.
